# Heat Shock Affects Amino Acid Metabolism in Bovine Cumulus Cells and Denuded Oocytes During In Vitro Maturation

**DOI:** 10.3390/biology15090682

**Published:** 2026-04-27

**Authors:** Hayder Radhi Hussein Mzedawee, Rasoul Kowsar, Golnaz Manian, Mehdi Hajian

**Affiliations:** 1 Department of Animal Science, College of Agriculture, Isfahan University of Technology, Isfahan 8415683111, Iran; h-mzedawee@ag.iut.ac.ir (H.R.H.M.); golnazmanian@ag.iut.ac.ir (G.M.); 2 Department of Animal Biotechnology, Reproductive Biomedicine Research Center, Royan Institute for Biotechnology, ACECR, Isfahan 8165131378, Iran

**Keywords:** amino acid metabolism, bovine cumulus cells, denuded oocyte, heat shock, in vitro maturation

## Abstract

Increased heat levels can affect follicle development, oocyte maturation, and bovine fertility by disrupting amino acid (AA) metabolism and oocyte competence. This study aimed to explore the effect of heat shock on AA metabolism in bovine cumulus cells (CCs) or denuded oocytes (DOs). CCs and DOs were separately cultured for 24 h in vitro at 38.5 °C (control group), 39.5 °C (moderate heat shock-CC, MHS-CC group), or 40.5 °C (high heat shock-CC, HHS-CC group). AA levels were analyzed in 24-h in vitro maturation media using high-performance liquid chromatography. The findings indicated that the HHS-CC group consumed more AAs than the control (*p* = 0.04) or MHS-CC group (*p* = 0.03). Compared with the control and HHS-CC groups, the MHS-CC group exhibited elevated alanine levels (*p* = 0.02). The MHS-CC (*p* = 0.03) and HHS-CC (*p* = 0.03) groups exhibited significantly greater glutamine depletion than the control group. The HHS-DO group exhibited significant lysine depletion (*p* < 0.01) but produced more tryptophan than the control and MHS-DO groups (*p* = 0.02). In contrast to the control and MHS-DO groups, the HHS-DO group displayed a notably elevated level of appearance (*p* = 0.005) and net balance (*p* = 0.005) for all AAs. The findings imply that heat shock may alter the metabolism of certain AAs in CCs and DOs, thereby affecting the developmental competence of bovine oocytes.

## Simple Summary

Heat shock disrupts normal energy metabolism in bovine oocytes and their surrounding cumulus cells, increasing metabolic stress. In response, both cell types adjust amino acid uptake and utilization to support survival, energy production, and redox balance. Amino acids such as glutamine, alanine, glycine, and branched-chain amino acids show altered turnover, reflecting their roles in mitochondrial metabolism, antioxidant defense, and osmotic regulation. Cumulus cells exhibit more pronounced metabolic flexibility, buffering the oocyte by supplying key amino acids and metabolic intermediates. Overall, changes in amino acid turnover represent an adaptive mechanism to maintain oocyte viability and developmental competence under heat stress.

## 1. Introduction

As climate change and global warming progress, animals are increasingly exposed to elevated temperatures and may experience reproductive issues [1]. This situation has emerged as a major challenge that hinders the future growth of livestock farming [1,2]. Lactating dairy cows are sensitive to heat stress, which diminishes oocyte quality for fertilization and their potential for further development [2,3]. It has been reported that heat stress leads to annual losses of approximately USD 1.5 billion [4], primarily due to seasonal infertility, declines in milk quality and production, and increased veterinary expenses [5,6,7].

Heat stress negatively affects oocyte quality in cows, hindering embryo development [2,8,9]. Oocytes are susceptible to temperature rises during maturation because heat stress speeds up the maturation process [10,11]. Heat-induced reductions in developmental competence after direct exposure of oocytes to 41 °C have been associated with reduced protein synthesis [12]. Therefore, the negative effects of heat shock may encompass germinal vesicle (GV)-stage oocytes [2], ovulated oocytes, zygote, and early cleavage-stage embryos [13]. All oocytes in the ovary are inhibited during the prophase stage of the meiotic cycle. The surrounding somatic cells produce substances that inhibit meiotic development during follicular expansion. At this point, the GV has a round-shaped nucleus with dispersed chromatin [14]. In vitro studies have shown that GV-stage cumulus-oocyte complexes (COCs) exposed to high temperatures have delayed nuclear and cytoplasmic maturation, increased aberrant spindle formation, and decreased developmental competence following in vitro fertilization [9,15]. Heat-stressed oocytes are halted at the metaphase I stage and fail to develop [9]. Furthermore, heat stress inhibits oocyte development in cows by affecting the production of progesterone, luteinizing hormone, and follicle-stimulating hormone (FSH) throughout the estrous cycle [16,17]. Heat stress may alter the hypothalamic-pituitary-ovarian axis, thereby reducing follicle growth and functioning [18]. These changes are associated with reduced steroidogenic ability of the dominant follicle, as evidenced by decreased estradiol [18,19] and increased plasma FSH concentrations [20]. Prolonged exposure to seasonal heat causes a suboptimal corpus luteum and lower plasma progesterone levels [17], resulting in lower embryo viability and early embryonic loss [21]. These hormonal changes decrease fertility by lowering oocyte quality, slowing follicular expansion, and producing poor embryos. In our previous study [22], COCs were matured at 38.5 °C, 39.5 °C or 40.5 °C and we found that heat shock reduced the quality, developmental competence, and altered AA metabolism in bovine COCs in vitro.

AAs influence oocyte physiology. AAs do more than serve as protein-building components. They are essential for energy metabolism (e.g., via TCA cycle intermediates), redox balance (glutamine feeds glutathione production), osmoregulation (glycine can function as an osmolyte), and signaling pathways that govern oocyte maturation, fertilization, and early embryo development [18,23,24,25]. Alanine is involved in transamination activities (e.g., linking AA metabolism to energy generation) and can transport nitrogen across cells [26]. Alanine depletion may indicate greater metabolic activity or stress in the oocyte [26]. Glutamine is an energy source, a precursor of nucleotide synthesis, and an antioxidant [18]. Glutamine depletion indicates either excessive consumption for redox balance or biosynthesis or inadequate supply from cumulus cells (CCs), which may affect oocyte quality [27]. Increased alanine and glutamine depletion may indicate that oocytes are under energy stress, which impairs ATP-dependent activities such as spindle formation, chromosomal segregation, and cytoskeletal dynamics [18,27]. Glutamine deprivation may diminish TCA cycle intermediates, thereby decreasing mitochondrial activity, which is a critical determinant of oocyte competence [18]. Furthermore, glutamine is a precursor of glutathione, the primary intracellular antioxidant. Lower glutamine levels may inhibit glutathione production, leading to increased oxidative stress, possible DNA damage or mitochondrial malfunction, and poor oocyte competence [18,27]. Therefore, alterations in AA depletion may interfere with oocyte maturation signaling, cumulus-oocyte communication, or early embryonic gene expression [18,27].

Embryo viability depends on proper metabolic and signaling support in the early stages of cleavage before implantation [28,29]. Within the COCs, the oocyte and the surrounding CCs exhibit co-dependence regarding both metabolic processes and signaling pathways [28,29]. CCs establish direct communication with the oocyte via transzonal projections that facilitate the transfer of regulatory factors and metabolites to the oocyte. When separated from the oocyte, CCs are unable to proliferate and expand and exhibit altered metabolic functions [28,29]. Without an oocyte, CCs lack metabolic competence, demonstrating disruptions in metabolism-related processes such as cumulus expansion [28,29]. Oocytes regulate the correct expression of genes, strongly promote DNA synthesis and cell growth, inhibit programmed cell death, support cellular differentiation, and enhance the transport of AAs in CCs [30]. Therefore, the oocyte plays a crucial role in guiding the differentiation and essential functions of CCs through the release of paracrine growth factors [31].

In addition, CC regulation directly influences oocyte development and their ability to support further embryo and fetal growth. Oocytes do not advance in meiosis when CCs are lacking [28]. Moreover, changes in metabolism can affect oocyte maturation by modulating CCs’ energy metabolism, proliferation, apoptosis, and steroid hormone synthesis [29]. *Slc38a3* mRNA expression is restricted to CCs in late antral follicles in mice [32], and it seems that intimate contact with the oocyte is necessary to improve AA transport cooperation between the two cell types, which is necessary to maintain oocyte growth [33]. For instance, oocytes lack the alanine transporter and depend on CCs to capture alanine [27]. Therefore, communication exists between the oocyte and adjacent CCs [32]. Therefore, the interaction between oocytes and CCs is vital for follicle development and optimum oocyte metabolism and competence [32,34].

The metabolic characteristics of oocytes and embryos can be evaluated using various techniques [35]. Laser scanning confocal microscopy and fluorescent lifetime microscopy (FLIM) are frequently employed to detect autoluminescent metabolic cofactors (such as NADH and FAD) [35]. Mitochondria in oocytes and embryos may be observed in three dimensions using confocal microscopy. FLIM offers information on the spatial distribution of metabolic cofactors in both the cytoplasm and mitochondria and has been effectively used to evaluate metabolic processes of oocytes [35]. Both FLIM and confocal microscopy will increase phototoxicity, which might delay or even fail blastocyst development [35]. Low-phototoxicity microscopy techniques, such as hyperspectral microscopy and two-photon excited fluorescence (TPEF) microscopy using a femtosecond laser are one potential option [35]. While hyperspectral microscopy uses a wide spectrum of light to detect a variety of endogenous fluorophores and provides a deeper understanding of oocyte and embryo metabolism, TPEF microscopy allows imaging at greater depths and lowers the risk of photodamage in out-of-focus areas [35,36]. Hyperspectral microscopy can distinguish between high- and low-quality bovine embryos [35,36]. As a result, it shows promise as a non-invasive method for identifying metabolic alterations linked to oocyte and embryo quality. Consequently, phototoxicity must be considered as a safety concern when using optical techniques. High-performance liquid chromatography (HPLC) is a simple and accessible technique for evaluating the metabolism of amino acids (AAs) as a non-invasive indicator for assessing the developmental potential of oocytes and embryos [23,24,26,37]. For instance, using the HPLC method, Hemmings et al. [23,26] found that oocytes with low developmental competence demonstrated significant AA turnover and consumed (depleted) a greater amount of AAs to fulfill their DNA repair requirements.

Crucially, the COC is a functional unit; yet, its functions may overlap or be difficult to discern if the oocyte and CCs are not kept apart. The oocyte and CCs may be studied separately after they are separated. Because oocyte-autonomous processes enable us to examine which changes in AA metabolism are oocyte-driven vs. cumulus-driven and vice versa, this may provide mechanistic insights. Thus, we hypothesized that heat shock may alter AA metabolism and that AA metabolism may follow distinct patterns in CCs and DOs.

## 2. Materials and Methods

All chemicals and media were sourced from Sigma-Aldrich Chemical Co. (St. Louis, MO, USA), unless otherwise specified.

### 2.1. Recovery of COCs Before In Vitro Maturation

The cow ovaries were transported from the slaughterhouse to the laboratory in a Thermobox maintained at approximately 30 °C. This box contained 0.9% sodium chloride solution along with 0.1% penicillin and streptomycin (Sigma-Aldrich, St. Louis, MO, USA). Upon arrival, the ovaries were rinsed in sterile 0.9% sodium chloride enriched with 0.1% penicillin and streptomycin at 30 °C and then preserved in sterile saline until they were aspirated using an 18G needle connected to a vacuum pump set at 80 mmHg. Only COCs with comparable dense CC layers were examined using a stereomicroscope (Olympus, Tokyo, Japan).

### 2.2. Oocyte Denudation and In Vitro Incubation

According to previous studies [38,39,40], oocytes were entirely denuded using manual pipetting using 0.1% (*w*/*v*) hyaluronidase in calcium-free PBS and subsequently washed in H-TCM-199 prior to maturation. We obtained 694 denuded oocytes (DOs), which were divided into three groups: 250 for the control (38.5 °C), 186 for the MHS-DOs (38.5 °C), and 258 for the HHS-DOs (40.5 °C). For 24 h, each group of 10 was matured in a specific medium under circumstances (50-µL droplets of maturation media) that were the same as the COC culture. The medium was collected and kept at −80 °C after the allotted time had elapsed. DOs were collected from three to five cows and randomly assigned to each experimental group at each of the six repetitions of this experiment (6 independent experiments with 2–3 replicates per independent experiment, resulting in a total of 17 replicates).

### 2.3. Collection and In Vitro Incubation of CCs

According to previous studies [38,41,42], after DOs were removed, CCs were washed twice by centrifugation (200× *g*, 5 min each). The pellets were then suspended in culture medium. The CCs were randomly divided into three groups: control, MHS-CCs, and HHS-CCs, maintaining a density of 2 × 10^5^ cells per ml for 24 h, identical to the COCs and DOs culture. The number of CCs cultured was determined based on the average count attached to each COC, approximately (2.3 ± 0.4) × 10^4^ cells. Thus, each drop included about 2 × 10^5^ CCs. CCs were collected from three to five cows and randomly assigned to experimental groups under conditions identical to the DO culture for 24 h in each of the four repetitions of this experiment (4 independent experiments with 2–3 replicates per independent experiment, resulting in a total of 10 replicates).

Maturation medium used for DOs and CCs culture consisted of TCM-199 enriched with Na pyruvate (2.5 mM), L-glutamine (1 mM), penicillin (100 IU/mL), streptomycin (100 µg/mL), fetal bovine serum (FBS; 10%, *v*/*v*), epidermal growth factor (EGF; 100 ng), follicle-stimulating hormone (FSH; 10 µg/mL), luteinizing hormone (LH; 10 µg/mL), estradiol-17β (1 µg/mL), and cysteamine (0.1 mM). The DOs or CCs were retrieved after the incubation period, and the maturation medium [six samples from the DO culture (6 independent experiments) and four samples from the CC culture (4 independent experiments)] was collected and stored at −80 °C for subsequent analysis.

### 2.4. Amino Acid Analysis

Droplets of maturation medium were collected individually to assess the levels of AAs following a 24-h incubation period. Following our previous research [38,43], the HPLC method (HyperClone ODS C 18, 250 mm, 4.6 mm, 5 m, Agilent 1100, Agilent Technologies, Waldbronn, Germany) was used to measure AAs in the 24-h spent medium (four samples from the 4 independent CC cultures and six samples from the 6 independent DO cultures) and in the fresh maturation media (n = 3). For the HPLC analysis, a flow rate of 1.2 mL/min was maintained, employing fluorescence detection after pre-column derivatization with o-phthalic-dialdehyde (OPA). The fluorescence signal was recorded at 450 nm when excited at 348 nm. The analysis procedure applied to all blanks and test media was consistent, with immediate storage at −80 °C. COC-free maturation media served as the control samples (blanks). Specifically, droplets of maturation medium without oocytes were cultured alongside the test samples at three distinct temperatures (38.5 °C, 39.5 °C, and 40.5 °C) for 24 h. 

To assess AA concentrations, the peak regions of the samples were analyzed alongside the reference AA combination. All peak signals were standardized using internal standards. The internal standards employed were 25 mM sarcosine and 25 mM norvaline, both dissolved in 0.1 M HCl. O-phthal-dialdehyde (OPA) has demonstrated a limited reaction with cystine and no reaction with proline in the presence of the thiol component, as indicated by the OPA technique utilized in this study [44]. Asparagine assay was unlikely due to the conversion of asparagine to aspartic acid during sample processing [45]. Phenylalanine (Phe), isoleucine (Ile), leucine (Leu), valine (Val), methionine (Met), threonine (Thr), lysine (Lys), arginine (Arg), tyrosine (Tyr), histidine (His), and tryptophan (Trp) were the EAAs in this study. Semi-EAAs were serine (Ser), glycine (Gly), and glutamine (Gln). Non-EAAs were aspartic acid (Asp), asparagine (Asn), alanine (Ala), citrulline (Cit), ornithine (Orn), and glutamic acid (Glu), according to previous studies [38,43,45,46]. In this study, we considered certain AAs, specifically Arg and Tyr, as EAAs despite their classification as semi-EAAs for cattle. Notably, certain cell types, such as CCs and oocytes, are unable to synthesize these AAs. Consequently, in this context, these AAs are considered essential [47].

### 2.5. Calculation of Amino Acid Depletion and Appearance

Previous research determined the depletion or appearance of AAs in picomoles per oocyte per hour [23,26,38,43]. The AA depletion or appearance was calculated based on the difference in AA concentrations between the fresh medium at the start of incubation (0 h) and the spent maturation medium after 24 h. Negative values observed after 24 h indicate “depletion,” signifying the loss of AAs from the maturation medium, whereas positive values indicate an increase in AA levels, referred to as “appearance.” The overall depletion and appearance of the AAs were quantified by aggregating the negative values (depletion) and positive values (appearance). To determine the “total turnover” of all AAs, the total depletion and total appearance were combined. The “total net balance” of all AAs was estimated by subtracting the total depletion from the total appearance.

### 2.6. Accuracy of Amino Acid Analysis

To assess the effectiveness of the AA analysis, we determined the average recovery from a biological medium containing a specific concentration of AAs corresponding to that found in the maturation medium. Accuracy was defined as the correlation between the measured and actual AA concentrations in a reference medium. The overall average recovery rate for all measured AAs was 102.6%. The precision varied between 93.1% and 106.9%, except for Asp, which had a precision of 112.4%. The acceptable range for all AAs was established at 90–115%. To further evaluate the precision of the method, we examined repeatability by injecting the same fresh maturation medium four times consecutively. This was accomplished by calculating the relative standard deviation (RSD) for each AA, with RSD values falling within an acceptable range of 1.12% to 3.36% [48].

### 2.7. Statistical Analysis

The Anderson-Darling test was used to assess the normality of the data distribution. In the case of being normal, Tukey’s multiple comparisons test was applied to analyze data. Conversely, for data that were not normally distributed, the Kruskal–Wallis test followed by Dunn’s multiple comparison test (nonparametric approach) was used. All *p*-values were adjusted using the Bonferroni correction method. In addition, the one-way ANOVA F-test confirmed the homogeneity of all data. All data are presented as mean ± standard error of the mean (SEM), and differences between groups were considered significant at *p* < 0.05. Analysis was performed using PAST and GraphPad Prism software (version 8.0).

## 3. Results

### 3.1. Heat Shock Affects the Depletion or Appearance of AAs by CCs During IVM

**Figure 1:**
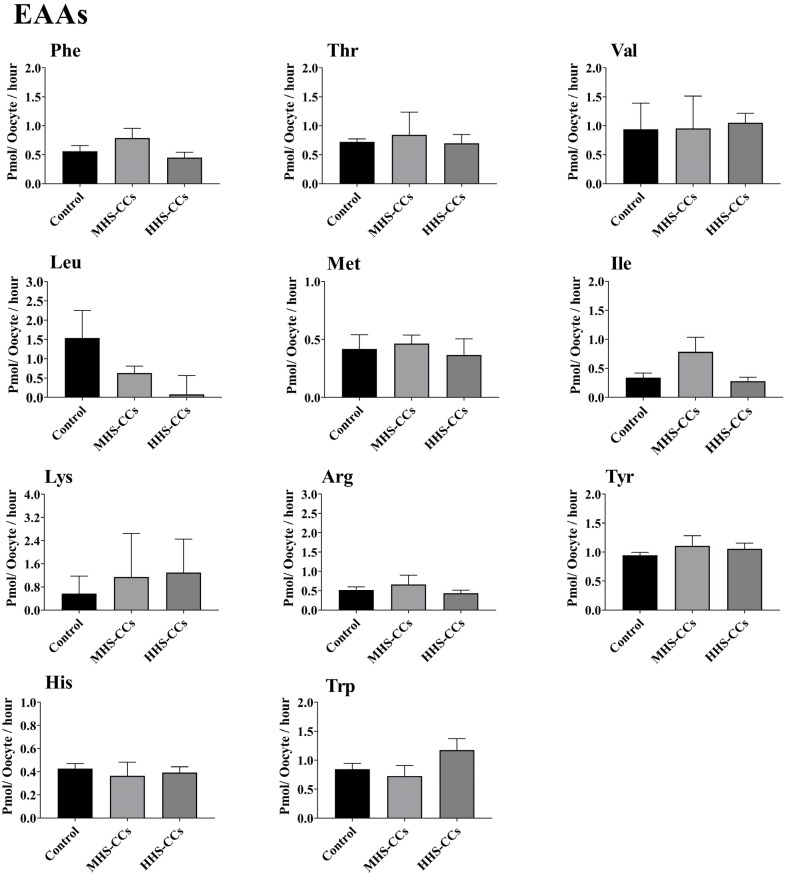
Effect of heat shock on the net depletion/appearance of essential amino acids (EAAs) by bovine cumulus cells (CCs) after 24 h in vitro maturation at 38.5 °C (control, n = 4 independent experiments), 39.5 °C (moderate heat shock-CCs, MHS-CCs, n = 4 independent experiments), or 40.5 °C (high heat shock-CCs, HHS-CCs, n = 4 independent experiments). Mean + SEM. Asterisks indicate significant differences between groups. In the case of existence of significant differences, * indicates *p* < 0.05. *p*-values were adjusted using Bonferroni correction method.

**Figure 2:**
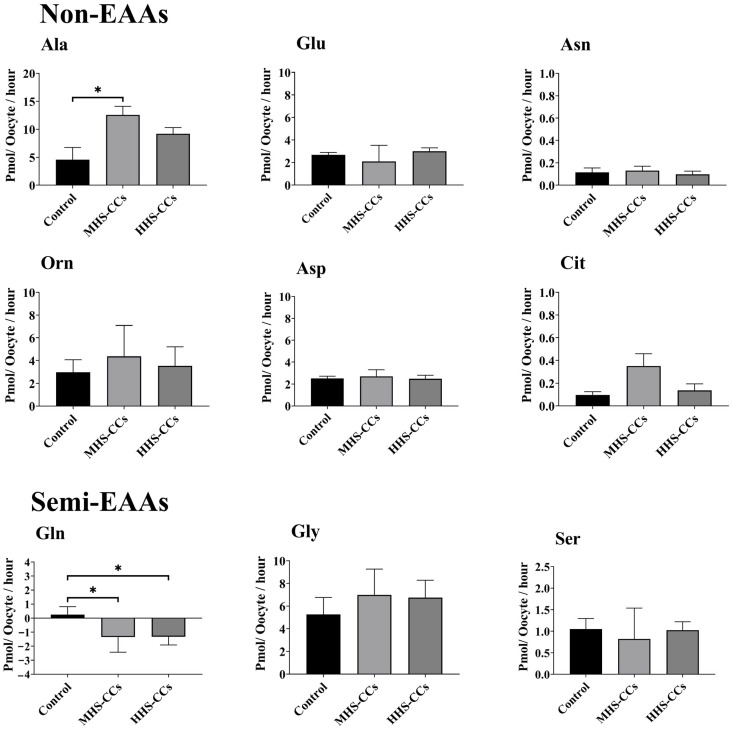
Effect of heat shock on the net depletion/appearance of non-essential amino acids (EAAs) and semi-EAAs in bovine cumulus cells (CCs) after 24 h in vitro maturation at 38.5 °C (control, n = 4 independent experiments), 39.5 °C (moderate heat shock-CCs, MHS-CCs, n = 4 independent experiments), or 40.5 °C (high heat shock-CCs, HHS-CCs, n = 4 independent experiments). Mean + SEM. Significant differences between groups are indicated by asterisks. * indicates *p* < 0.05. *p*-values were adjusted using Bonferroni correction method.

**Figure 3:**
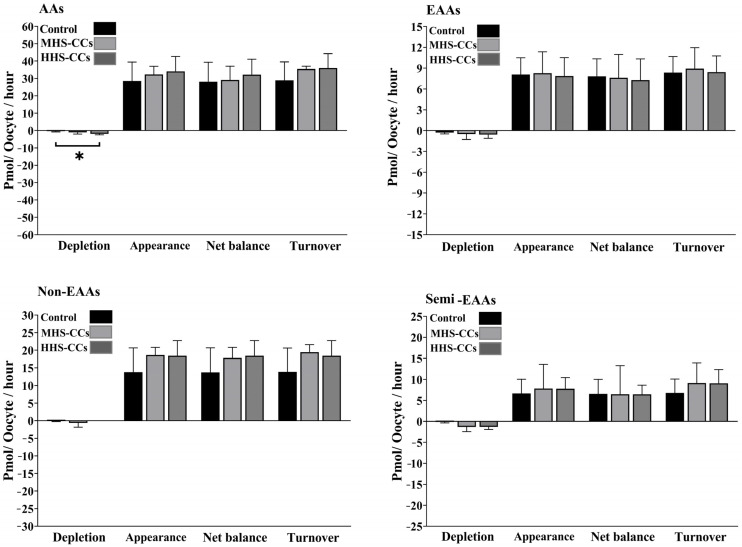
Effect of heat shock on the total depletion, appearance, net balance, and turnover of all amino acids (AAs), essential AAs (EAAs), non-EAAs, and semi-EAAs in bovine cumulus cells (CCs) after 24 h in vitro maturation at 38.5 °C (control, n = 4 independent experiments), 39.5 °C (moderate heat shock-CCs, MHS-CCs, n = 4 biological samples), or 40.5 °C (high heat shock-CCs, HHS-CCs, n = 4 independent experiments). “Total depletion” or “total appearance” of AAs means the sum of all negative or positive values obtained for non-EAAs. The “net balance” of AAs was calculated by subtracting depletion from appearance, and the AA turnover was the sum of net depletion and appearance. Mean + SEM. Asterisks indicate significant differences between groups. * indicates *p* < 0.05. *p*-values were adjusted using Bonferroni correction method.

Heat shock had no effect on the appearance (*p* = 0.45) or depletion (*p* = 0.51) of EAAs in CCs (Figure 1). The MHS-CC group produced greater amounts of Ala (*p* = 0.02, Figure 2). Among semi-EAAs, Gln depletion was higher in the MHS-CC and HHS-CC groups (*p* = 0.03) than in the control group (Figure 2). The total depletion of all AAs was greater in the HHS-CC group than in the control group (*p* = 0.03, Figure 3).

### 3.2. The Appearance and Depletion of AAs by DOs During IVM Are Affected by Heat Shock

**Figure 4:**
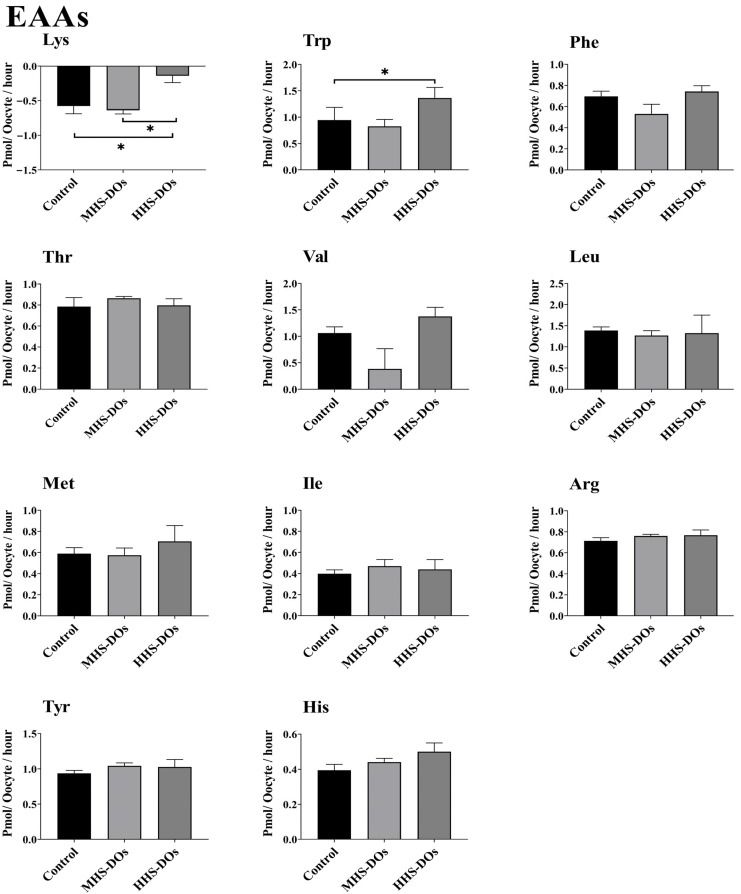
Effect of heat stress on the net depletion/appearance of essential amino acids (EAAs) in bovine denuded oocytes (DOs) after 24 h in vitro maturation at 38.5 °C (control, n = 6 independent experiments), 39.5 °C (moderate heat shock-DOs, MHS-DOs, n = 6 independent experiments), or 40.5 °C (high heat shock-DOs, HHS-DOs, n = 6 independent experiments). Mean + SEM. Significant differences between groups are indicated by asterisks. * indicates *p* < 0.05. *p*-values were adjusted using Bonferroni correction method.

**Figure 5:**
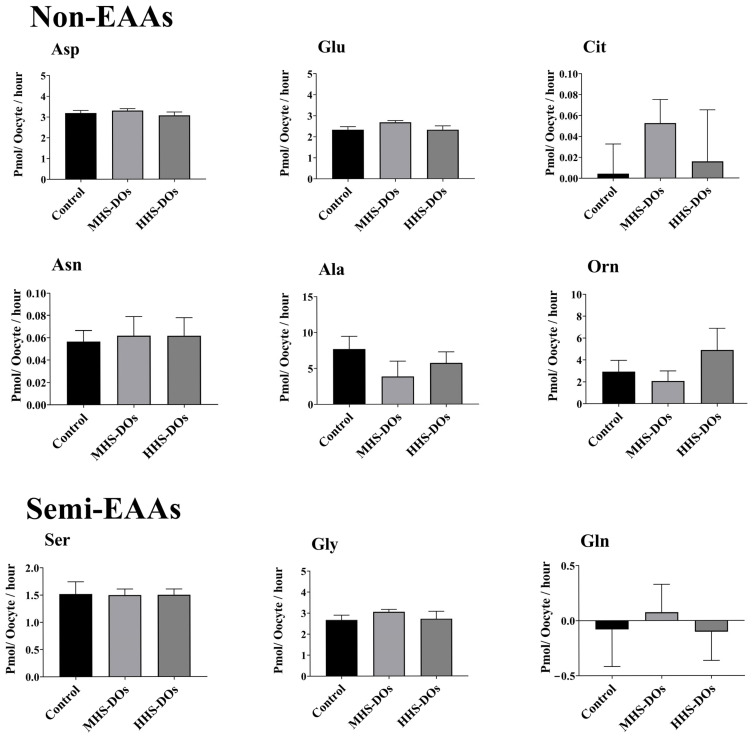
Effect of heat shock on the net depletion/appearance of non-essential amino acids (EAAs) and semi-EAAs in bovine denuded oocytes after 24 h in vitro maturation at 38.5 °C (control, n = 6 independent experiments), 39.5 °C (moderate heat shock-DOs, MHS-DOs, n = 6 independent experiments), or 40.5 °C (high heat shock-DOs, HHS-DOs, n = 6 independent experiments). Mean + SEM. In the case of existence of significant differences, * indicates *p* < 0.05. *p*-values were adjusted using Bonferroni correction method.

Compared with the control and MHS-DO groups, the HHS-DO group exhibited increased Trp production (*p* = 0.02) and lesser Lys depletion (*p* < 0.01, Figure 4). Heat shock had no effect on the appearance or depletion of non-EAAs or semi-EAAs in DOs (Figure 5).

### 3.3. AAs’ Appearance/Depletion by DOs

**Figure 6:**
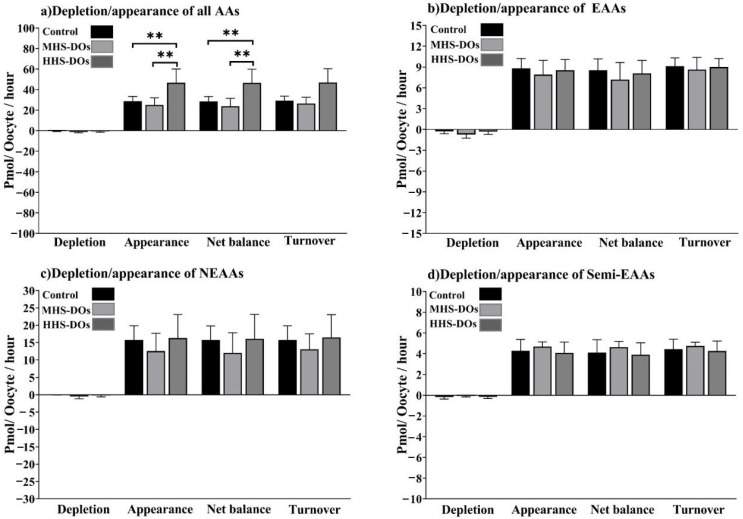
Effect of heat shock on the total depletion, appearance, net balance, and turnover of all amino acids (AAs), essential AAs (EAAs), non-EAAs, and semi-EAAs in bovine denuded oocytes (DOs) after 24 h in vitro maturation at 38.5 °C (control, n = 6 independent experiments), 39.5 °C (moderate heat shock-DOs, MHS-DOs, n = 6 independent experiments), or 40.5 °C (high heat shock-DOs, HHS-DOs, n = 6 independent experiments). “Total depletion” or “total appearance” of AAs means the sum of all negative or positive values obtained for non-EAAs. The “net balance” of amino acids was calculated by subtracting depletion from appearance, and the AA turnover was the sum of net depletion and appearance. Mean + SEM. Asterisks indicate significant differences between groups: ** indicates *p* < 0.01. *p*-values were adjusted using Bonferroni correction method.

The HHS-DO group outperformed the control and MHS-DO groups in terms of the appearance of all AAs (*p* = 0.005) and net balance (*p* = 0.005; Figure 6).

## 4. Discussion

This study implied that heat shock may differentially change AA metabolism in bovine CCs and DOs in vitro. Amino acid metabolism in oocytes is closely linked to the use of carbohydrates and fats, especially when heat stress compromises energy homeostasis and redox balance [49,50,51]. Heat stress reduces glycolytic flow and mitochondrial efficiency, leading to compensatory changes in substrate use [49,50,51]. Decreased glycolytic capability might make it more dependent on lipid β-oxidation to maintain ATP synthesis, which would raise acetyl-CoA and NADH levels and affect AA catabolism and mitochondrial transamination events [49,50,51,52]. Concurrently, when carbohydrate-derived inputs are scarce, increased AA turnover, particularly of glucogenic and anaplerotic AAs, may sustain mitochondrial activity by replenishing TCA cycle intermediates [50,51,52]. Furthermore, by connecting their metabolism to redox adaptation under heat stress, AAs including glutamine, glycine, and serine may support osmoprotection and antioxidant defense [50,51,52]. Therefore, a coordinated metabolic reprogramming in which AA turnover buffers disruptions in glycolysis and lipid metabolism, ultimately supporting oocyte survival and developmental competence under elevated temperature, is probably responsible for the altered AA profiles seen in heat-stressed oocytes.

Previous studies have documented the significance of CCs in oocyte maturation and developmental competence establishment [53]. The metabolic states of DOs and CCs were examined as they matured at three different temperatures. According to our findings, a high amount of Ala appeared in the medium when the CCs were exposed to 40.5 °C during IVM. Oocytes from cows that did not cleave produced more Ala than those that did [26]. CCs, which are essential in this process, take up certain AAs, such as Ala, which are poorly transported into oocytes. The CCs should first take up Ala and then transfer to the oocyte by the gap junctions. Ala plays a functional role in protecting cells from stress-induced damage and promoting the expression of genes involved in stress protein production [54]. Therefore, the current study concluded that oocytes might be Ala-deficient, as shown by the lower developmental competence and quality of COCs. Therefore, the higher Ala levels in the HHS-CCs group in the current study may be explained in part by the reduced Ala uptake caused by heat shock-decreased CC viability. This study showed that the HHS-CC and MHS-CC groups depleted more Gln than the control group. Purines and pyrimidines are required for DNA repair [55] and can be produced via Gln [56], indicating that oocytes that cannot cleave have a higher requirement for Gln. High temperatures cause oocytes to produce more reactive oxygen species (ROS), which increases oxidative stress and metabolic activity [57]. Heat stress changes the function of antioxidant enzymes in cells, making oocytes more vulnerable to oxidative damage and affecting embryonic development [13]. Energy generation, calcium homeostasis, and oocyte apoptosis all depend on mitochondrial activity, which can be compromised by high temperatures [50]. The mitochondrial activity of both immature and mature oocytes is decreased by heat shock, which has a direct effect on their ability to grow [50]. Heat stress causes an increase in ROS, which damages mitochondria during oocyte maturation and lowers developmental competence [50,51]. DNA breakage and even cell death can result from cytoplasmic disruptions brought on by high temperatures, which are similar to cell aging [58]. This finding implies that heat shock may increase Gln uptake by CCs to ensure CC survival and DNA repair.

According to our study, the HHS-CC group consumed more AAs overall than either the control or MHS-CC groups. Incompetent oocytes exhibited greater overall depletion of all AAs than their cleaved counterparts [23,26]. Thus, it is possible to propose that heat shock decreases oocyte competence by increasing the demand for AAs and causing complete AA depletion.

The HHS-DO group had a greater level of appearance and net balance for all AAs than the control and MHS-DO groups. The appearance of AAs is correlated with oocyte quality [23,26]. Uncleaved oocytes may show noticeably higher AA appearance than cleaved oocytes [23,26]. According to our results, HHS-DO appeared to have higher Trp and less Lys depletion than the control or MHS-DO groups. L-Lys supplementation at doses ranging from 0.5 to 5.0 mmol/L considerably enhanced the vitality of bovine epithelial cells in the mammary gland compared with cells without L-Lys supplementation [59]. In bovine mammary epithelial cells, Lys activates CDK1 expression by activating the ERK signaling pathway via SLC6A14 [60]. In bovine mammary epithelial cells, CDK1 can stimulate β-casein production and proliferation via the mTOR signaling pathway [60]. For decades, Trp has been the focus of scientific studies in human biology due to its ability to change into a number of tiny, pleiotropic, bioactive molecules that can affect various physiological reactions and cell metabolic pathways after absorption. Therefore, changes in L-Trp-derived compounds have been linked to a number of metabolic disorders and syndromes that impact the organs and systems responsible for preserving chemical, cellular, and behavioral homeostasis, including the immune, neuroendocrine, and gut-liver apparatus as well as the central nervous system. During development, an unbalanced metabolism of AA can disrupt the capacity of these systems to interact and differentiate between stressors and stimuli, exogenous and endogenous antigens, nutrients, and xenobiotics [61].

The metabolic codependence of CCs and oocytes has been well established [31,62,63]. Small regulatory factors and metabolites can physically reach the follicle through CCs, acting as a biological barrier between the oocyte and its environment. CCs deal with substrates, such as glucose, that the oocyte cannot or is not able to metabolize [64]. Moreover, CCs shield the oocyte from oxidative stress by increasing the amount of glutathione in the oocytes or producing the antioxidant catalase [65]. CCs use glycolysis to produce lactate and pyruvate, aiding meiotic maturation to the metaphase II (MII) stage of oocytes [28]. Oocytes exhibit disrupted metabolism and are less able to sustain fertilization and subsequent embryonic development when surrounding CCs are absent during IVM [28]. CCs deliver energy substrates, including fatty acids, carbohydrates, and AAs, from the surrounding fluids to the oocyte. By secreting various oocyte-specific factors, the oocyte also helps CC function by controlling its microenvironment and improving its ability to develop into an embryo [66]. Some cellular metabolism-related genes are expressed differently in bovine COCs and DOs following IVM in supplemented-serum medium [67]. These observations support the idea that CCs play a significant role in the appropriate control of oocyte metabolism during IVM [68]. For both compartments to mature and function, oocytes and partner CCs must be able to communicate in both directions. Oocytes lack the capacity to perform glycolysis, produce cholesterol, and transport certain AAs. CCs supply the particular AAs and products involved in these metabolic pathways [38]. Oocytes regulate metabolic activity in CCs by promoting the expression of genes encoding AA transporters and enzymes necessary for oocyte-deficient metabolic processes [69]. CCs are assigned metabolic functions to compensate for metabolic inadequacies. Through endocrine, paracrine, and autocrine mechanisms, the oocyte may regulate its CC metabolism and change follicular growth rate [65,69]. Although juxtacrine signals most likely also play a role, paracrine factors are the primary way that oocytes affect the development of granulosa cells. Growth differentiation factor-9, bone morphogenetic protein-15, and fibroblast growth factor-8b are important oocyte-derived paracrine proteins [70]. These findings imply that the presence of CCs may affect the metabolism of AA in DOs under heat shock.

However, in this study, we focused on AA metabolism changes by heat stress in DOs and CCs; AA metabolism in oocytes or CCs may be linked to oocyte developmental competence or embryo outcome. Aneuploid embryos have been shown to have a negative increase in AA turnover compared with normal embryos [71]. Embryos that were halted 2–3 days after fertilization had a higher turnover of AAs than those that progressed to the blastocyst stage [24]. Furthermore, there was a significant AA turnover in oocytes with low developmental ability. In fact, uncleaved oocytes may consume (deplete) more resources, such as AAs to satisfy their demands for DNA repair [26]. In a prior work, our team found that urea dramatically altered the AA turnover by COCs, CCs, and DOs and decreased the future developmental competence of bovine oocytes [38]. Furthermore, we recently documented that heat shock during IVM negatively impacted the quality and developmental competence of bovine COCs and the AA metabolism [22]. We discovered a strong correlation between developmental capability of bovine COCs and AA metabolism (Orn, Glu, and Ala) during IVM [22]. The mRNA expression of *OCT-4*, *IGF-2R*, *GDF-9*, *MnSOD*, and *GLUT-1* was reduced throughout the hot period in buffalo MII oocytes and 2-cell embryos, according to a previous study [72]. Harmful seasonal effects during the GV stage might affect the quality of formed blastocysts and oocyte developmental competency in later stages of embryonic development [72]. Therefore, heat shock is thought to affect AA metabolism in bovine COCs, DOs, and CCs during IVM, which would lower oocyte competence and early embryo development.

## 5. Limitations

There were some limitations to this study. First, in vitro and physiological follicular settings differ from one another. In the absence of any significant meiotic inducers, the majority of oocytes will spontaneously resume meiosis and attain MII when a COC is removed and cultured in vitro because the ovarian follicular environment maintains oocyte meiotic arrest [30]. Next, in contrast to the long-term in vivo heat stress, we used a restricted model of a 24-h heat exposure in this study. Furthermore, because we cultivated DOs in groups of 10, cellular heterogeneity may be obscured by the lack of single-cell resolution. Furthermore, this study did not assess direct assays (such as MitoSOX, JC-1, or ATP measurement) associated with free radical formation during heat stress. Crucially, the relationship between heat shock and altered metabolism is still hypothetical in the absence of such evidence. Although there is no molecular confirmation, the current study provided quantitative data on AA depletion and appearance. Thus, future research must incorporate proteomic or gene expression data (such as the expression of pertinent AA transporters (SLC family) or stress-response genes, such as *HSP70*, *NRF2*, and *SLC38A*). Finally, additional significant stress-related metabolites, such as taurine or cysteine, were not included in the limited AA panel (17 AAs).

## 6. Conclusions

According to this study, heat stress may alter AA metabolism differently in CCs and DOs. Because oocytes have more mitochondria than any other cell type, mitochondrial control is essential for the metabolic response to heat stress [73]. By upsetting the electron-transport chain, decreasing ATP synthesis, and raising the generation of reactive oxygen species, elevated temperatures can harm mitochondrial function [50,51]. Under such stress, both nucleus-encoded and mitochondrially encoded mitochondrial protein genes frequently alter their expression patterns, affecting the metabolism of carbohydrates, lipids, and AAs [74,75]. This may suggest potential variations in mitochondrial gene expression between heat-stressed oocytes and cumulus cells. The different metabolic activities observed in these two cell types may be explained by this variation. Additionally, recent research has demonstrated that the expression of mitochondrial metabolic genes varies by tissue [74,75]. Nevertheless, how heat shock influences the metabolism of AAs remains unknown.

## Data Availability

The data underlying this article are available in the article.
